# Climate change and fluid status in children: early education as one response to an emerging public health problem

**DOI:** 10.1017/S1368980023002562

**Published:** 2023-12

**Authors:** Hermann Kalhoff, Kathrin Sinningen, Aziza Belgardt, Mathilde Kersting, Thomas Luecke

**Affiliations:** 1 Pediatric Clinic Dortmund, Beurhausstrasse 40, D-44137 Dortmund, Germany; 2 Research Department of Child Nutrition, University Hospital of Pediatrics and Adolescent Medicine, St. Josef-Hospital, Ruhr-University Bochum, Alexandrinenstrasse 5, D-44791 Bochum, Germany

**Keywords:** Global warming, heat stress, children, fluid balance, educational concepts

## Abstract

**Objective:**

As global warming intensifies, residents of temperate regions will also face heat waves in the near future. Food habits are one component in addressing the global challenge of climate change. However, water, the most important food for humans, has not been adequately addressed.

**Design:**

For this commentary, on the one hand, publications on the increasing heat stress of children were consulted. On the other hand, publications on the special demands of children’s temperature regulation in hot environments on fluid balance were analysed.

**Setting:**

The situation of young children in care facilities on days with heat stress is presented as a scenario. In this way, the effects of climatic changes on fluid balance can be estimated and measures to reduce heat stress and stabilise the fluid balance of children can be developed.

**Participants:**

For this analysis, first, infants will be considered in order to identify their specific fluid needs. Second, the possibilities for caregivers to improve fluid intake and train appropriate drinking habits already in infancy will be highlighted.

**Results:**

Climate change should be included in recommendations on hydration for children. The need to adapt drinking habits requires educational approaches to weather and water – starting in early childhood care.

**Conclusions:**

In the face of rapid climate change, countries must act now by protecting, preparing and prioritising the high-risk group of children. Particular focus should be placed on supporting adequate hydration.

## Climate change – risk prognosis for children

### Increasing risk of heat waves

Global climate change with rising average temperatures is the central threat worldwide to our environment and thus also to the health of the population. About two billion people worldwide do not have access to clean drinking water today, and about half of the world’s population suffers from severe water scarcity for at least part of the year. These numbers are expected to rise due to climate change and population growth^(1)^. As global warming increases, even residents of temperate latitudes will face overheating and heat waves in the coming years and decades. Global warming will have a significant impact on the quality of life and sustainability (such as reducing emissions, expanding the share of renewable energy, and optimising the circular economy), especially in cities^(1,2)^.

Already, about 559 million children are affected by frequent heat waves, and about 624 million children are exposed to one of three other heat measures: duration of heat wave, severity of heat wave, or extremely high temperatures. By 2050, virtually every child on Earth – more than 2 billion children – is expected to be exposed to more frequent heat waves. Children in northern regions will face the most dramatic increase in heat waves, while nearly half of all children in Africa and Asia will be permanently exposed to extremely high temperatures by 2050^(3)^. These findings underscore the urgent need to adapt the services children rely on as unavoidable impacts of global heating unfold.

### Climate change and lifestyle

Since climate change is largely determined by current lifestyles, a fundamental paradigm shift in the assessment of current lifestyles is necessary for risk reduction. Globally, goals have been formulated, for example, in the UN’s Sustainable Developmental Goals and in the guidelines of the Paris Climate Change Agreement^(4)^.

### Eating and drinking habits

Dietary habits are an important component in the actions to address the global challenge of climate change for future generations. Specifically, this challenge for food habits means that a shift to a plant-based diet and sustainable food production is necessary^(5)^. However, water, the most important food for humans, has not been sufficiently considered in terms of the required dietary change.

## Fluid balance and temperature regulation

### Water: physiological requirement

The human body consists of a considerable amount of water. This involves orders of magnitude from 70–80 % in newborns to 40–50 % in adults and 35 % in the elderly. The daily turnover rate of water is higher than that of any other nutrient, ranging in magnitude from one-sixth of body weight per d in infants to about one-fortieth in adults^(6,7)^. Even small water deficits lead to functional impairments, whereas increased drinking of water in children can, for example, promote cognitive performance^(8)^.

### Water and child health

Children are a particularly vulnerable group when it comes to protecting their health. For example, the regulation of water balance in the body is particularly susceptible to disruption. On the one hand, children need more water than adults in relation to their body weight. Both an insufficient quantity and an insufficient quality of water endanger the health and development of children. This is all the more true for the youngest children. Literature data suggest that children in Europe, for example, already consume slightly less fluid than the recommended amount on average every day^(9)^.

### Temperature regulation in heat

The rate of heat storage in the body (S) can be described as the difference between the metabolic rate (M), the external work rate (Wk), the rates of heat transfer by conduction (K), radiation (R), convection (C) and evaporation (E) from the skin surface, and the rates of heat transfer by convection and evaporation from the respiratory tract (Cres and Eres), thus:

S = M - Wk - K - R - C - E - (Cres + Eres)^(10)^.

**Fig. 1:**
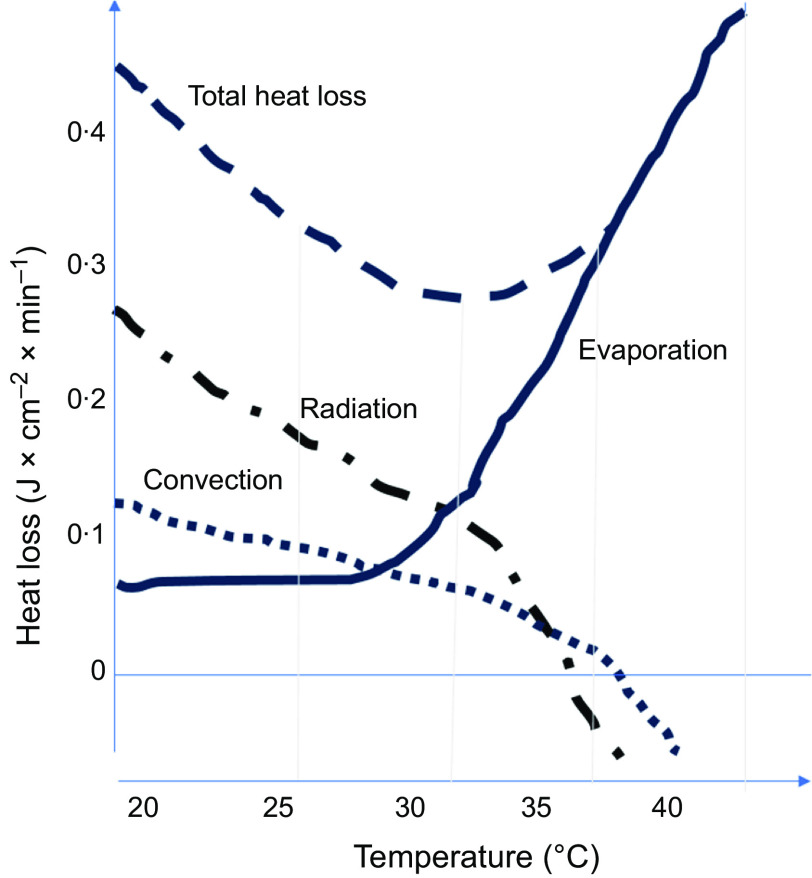
Heat transfer by radiation, convection and evaporation at different room temperatures ^(modified after^ ^11)^

As the ambient temperatures increase, the possibility of heat transfer via the processes of radiation and convection decreases. From about 35 degrees, effective heat loss to avoid overheating of the body is only possible through sweating (Fig.1).

In relation to their body weight, children have a much larger body surface area than adults. In contrast, the capacities for active counter-regulatory processes (often as a function of body mass) are still relatively low. Therefore, children have an increased risk of overheating in heat waves, particularly when the ambient temperature exceeds the skin temperature at over 30 degrees Celsius^(10)^.

### Fluid balance in heat

At high ambient temperatures, the amount of water needed to compensate for temperature via sweating increases sharply in addition to other requirements. This means that as the climate warms, the risk of water loss through the skin surface is high. As the climate warms, and particularly during heat waves, it is essential to adapt drinking habits to maintain health and performance, especially for children. Thus, the challenges of global warming should be considered in hydration recommendations for children.

### Water as the regular choice of beverage

From the point of view of health care and the prevention of later diet-related diseases, water is an exceptionally healthy beverage: water is calorie-free and therefore generally the first choice for meeting fluid requirements. This is even more true when the need for fluids increases, for example, as a result of global warming. If this necessary high fluid intake is covered by drinking water, this also makes a significant contribution to sustainable nutrition from a climate protection point of view, especially if tap water is drunk. For example, the CO2 footprint when using (packaged) mineral water is many times higher compared to tap water, depending on the consideration of the individual process steps. Taken together, this results in a clear multi-perspective chain of arguments for drinking water as a regular beverage from early childhood.

However, water scarcity through climate change and the resulting increase in the costs of water can lead to inequitable access. This may deprive households of opportunities to collect the amount of safe water needed for proper handwashing and hygiene, limiting children’s ability to grow up healthy and strong. Moreover, there is a cost in terms of household income and plant health in terms of packaging and transporting water^(1,3)^.

## Early Education for children

### Global warming and drinking habits

An adjustment of the drinking habits is indispensable for the maintenance of health and efficiency. This will require developing environmentally friendly drinking recommendations for children that meet physiological water needs in different weather scenarios, especially in light of increasing global warming and more frequent heat waves. The fact that the messages about drinking water are positive, as opposed to restrictions or even bans (on alternative drinks that are popular with children, such as soft drinks and juices), increases the prospects for success.

### Promoting drinking water in daycare centres: setting-based approach

Early education is a useful starting point when it comes to long-term acquisition of health-promoting behaviours. Learning to drink water regularly at an early stage can support a healthy lifestyle. Changing drinking habits as an adaptation to global warming should be taught to children as early as possible, that is, in daycare centres and kindergardens (e.g. as part of the concept of a ‘healthy daycare centre’). For this purpose, pedagogical concepts on the topic of weather and water should be developed for early childhood care. The importance of adequate hydration should be continuously taught, starting from early childhood. Because any type of behaviour change is challenging^(12)^, multiple approaches are needed in addition to setting-based approaches, such as home-based approaches, public health strategies, social norms or networking with community-based services.

In the face of rapid climate change, countries must act now by protecting, preparing and prioritising the high-risk population of children.

## References

[1] IPCC (2023) Climate change 2023: synthesis report. In *Contribution of Working Groups I, II and III to the Sixth Assessment Report of the Intergovernmental Panel on Climate Change*, pp. 35–115 [Core Writing Team, H Lee and J Romero , editors]. Geneva, Switzerland: IPCC. doi: 10.59327/IPCC/AR6-9789291691647.

[2] Nazarian N , Krayenhoff ES , Bechtel B et al. (2022) Integrated assessment of urban overheating impacts on human life. *Earth’s Future* 10, e2022EF002682. doi: org/10.1029/2022EF002682.

[3] United Nations Children’s Fund (2022) The Coldest Year of the Rest of their Lives: Protecting Children from the Escalating Impacts of Heatwaves. New York: UNICEF.

[4] United Nations (2015) Sustainable Developmental Goals Transforming Our World: The 2030 Agenda for Sustainable Development. https://sdgs.un.org/2030agenda (accessed September 2023).

[5] Eat Lancet Commission (2019) Food in the Anthropocene: the EAT–Lancet Commission on Healthy Diets from Sustainable Food Systems. https://www.thelancet.com/commissions/EAT (accessed August 2023). 10.1016/S0140-6736(18)31788-4 30660336

[6] Manz F , Wentz A & Sichert-Hellert W (2002) The most essential nutrient: defining the adequate intake of water. J Pediatr 141, 587–592. doi: 10.1067/mpd.2002.128031. 12378203

[7] Kalhoff H , Hilbig A & Libuda L (2015) Fluid intake – what to drink and how much? Physiology and practice from infancy to adolescence [Article in German]. Kinder- Jugendmedizin 15, 7–12.

[8] Drozdowska A , Falkenstein M , Jendrusch G et al. (2020) Water consumption during a school day and children’s short-term cognitive performance: the CogniDROP randomized intervention trial. Nutrients 12, 1297 doi: 10.3390/nu12051297. 32370147 PMC7282257

[9] Iglesia I , Guelinckx I , De Miguel-Etayo PM et al. (2015) Total fluid intake of children and adolescents: cross-sectional surveys in 13 countries worldwide. Eur J Nutr 54, 57–67. doi: 10.1007/s00394-015-0946-6. 26081646 PMC4473088

[10] Cramer MN , Gagnon D , Laitano O et al. (2022) Human temperature regulation under heat stress in health, disease, and injury. Physiol Rev 102, 1907–1989. doi: 10.1152/physrev.00047.2021. 35679471 PMC9394784

[11] Persson PB. (2019) Energy and heat balance, Thermoregulation (article in German). In Physiologie des Menschen, 32nd ed., pp. 535–550 [ R Brandes , F Lang and RF Schmidt , editors]. Berlin-Heidelberg: Springer.

[12] Scaglioni S , De Cosmi V , Ciappolino V et al. (2018) Factors influencing children’s eating behaviours. Nutrients 10, 706. doi: 10.3390/nu10060706. 29857549 PMC6024598

